# Long-Acting Injectable Second-Generation Antipsychotics in Seriously Ill Patients with Schizophrenia: Doses, Plasma Levels, and Treatment Outcomes

**DOI:** 10.3390/biomedicines12010165

**Published:** 2024-01-12

**Authors:** Juan José Fernández-Miranda, Silvia Díaz-Fernández, Francisco Javier Cepeda-Piorno, Francisco López-Muñoz

**Affiliations:** 1 Cabueñes Universitary Hospital, Asturian Health Service (SESPA), 33394 Gijón, Spain; silvia.diaz@sespa.es (S.D.-F.); franciscojavier.cepeda@sespa.es (F.J.C.-P.); 2 Asturian Health Research Institute (ISPA), 33011 Oviedo, Spain; 3 Health Sciences Faculty, Camilo José Cela University, 28692 Madrid, Spain; flopez@ucjc.edu; 4 Neuropsychopharmacology Unit, 12 de Octubre Hospital Research Institute, 28041 Madrid, Spain

**Keywords:** schizophrenia, clinical severity, long-acting injectable antipsychotic, paliperidone, aripiprazole, dose, plasma levels, treatment outcomes, tolerability, compliance

## Abstract

This research studies the dose–plasma level (PL) relationship of second-generation antipsychotics, together with the treatment outcomes achieved, in seriously ill people with schizophrenia. An observational, prospective, one-year follow-up study was carried out with patients (N = 68) with severe schizophrenia treated with paliperidone three-month (PP3M) or aripiprazole one-month (ARIM). Participants were divided into standard-dose or high-dose groups. PLs were divided into “standard PL” and “high PL” (above the therapeutic reference range, TRR) groups. The dose/PL relationship, and severity, hospitalizations, tolerability, compliance, and their relationship with doses and PLs were evaluated. There was no clear linear relationship between ARIM or PP3M doses and the PLs achieved. In half of the subjects, standard doses reached PLs above the TRR. The improvements in clinical outcomes (decrease in clinical severity and relapses) were related to high PLs, without worse treatment tolerability or adherence. All participants remained in the study, regardless of dose or PL. Clinical severity and hospitalizations decreased significantly more in those patients with high PLs. Considering the non-linear dose–PL relationship of ARIM and PP3M in people with severe schizophrenia, PLs above the TRR are linked to better treatment outcomes, without worse tolerability. The need in a notable number of cases for high doses to reach those effective PLs is highlighted.

## 1. Introduction

Antipsychotic medications (APs) are an effective treatment for schizophrenia for patients who need continuous long-term treatment [1,2,3,4,5,6,7]. The high prevalence of non-adherence is associated with relapses and hospital (re)admissions [8,9,10,11] and suicide risk [12,13,14]. Second-generation antipsychotics (SGAs) have shown to be more effective and tolerable than first-generation antipsychotics (FGAs) [15,16]. While the use of SGA has already been recommended worldwide [5,6,11,16], there is a debate about the use of long-acting injectable (LAI) APs to improve adherence and treatment outcomes compared to oral APs [17,18,19,20,21]. LAI APs are considered an effective treatment strategy for improving compliance [9,11,18,20] and reducing psychiatric hospitalizations [22,23,24,25,26], being more effective than oral APs [5,7,17,18,19,20,22,23,24,25,26], especially in patients with poor compliance and relapses [20,27,28,29]. Observational data do not suggest significant differences between aripiprazole LAI and paliperidone LAI regarding the main clinical outcomes (hospitalization, symptom improvement, discontinuation) in head-to-head real-world comparisons [30,31].

The effectiveness of APs is related to the achievement of optimal plasma levels (PLs) [32,33,34,35]. Indeed, the quantification of PLs (“pharmacotherapeutic monitoring”) is considered a way for precision medicine to guide routine practice and improve treatment decisions [36,37,38,39]. Nevertheless, its usefulness in clinical practice is not clear, given the interindividual variability in the pharmacokinetics of APs, and in the dose–PL–response relationship [40,41,42].

An issue that is also discussed is the use of high doses of APs (those that exceed the recommended/licensed) in complex, seriously ill, or treatment-resistant patients. Although clinical guidelines state that there is no reason for the use of high doses [43,44], there are some arguments related to individual patient pharmacokinetic differences supporting the rationale behind their use [45,46]. What is certain is that high doses of APs in the treatment of schizophrenia are often prescribed because of the poor response to standard treatment [47,48,49,50,51]. Conversely, there is little to support pharmacodynamic differences as an explanation for the use of high doses [45,46,49,50,51]. Moreover, robust data on the adverse effects of high doses are still lacking [45,46,47,48,49,50,51].

These reasons support that it is necessary to know the PLs achieved with the different doses and their relationship with effectiveness, compliance, tolerability, and safety, especially in patients with poor progress. This study researches the dose–PL relationship of the most recently marketed antipsychotics in their long-acting injectable formulations: aripiprazole one-month (ARIM) [52,53,54] and paliperidone three-month (PP3M) [55,56,57,58]. The objectives here are to find the relationship between the doses prescribed in clinical practice and the PLs of ARIM and PP3M in severely ill people with schizophrenia; to ascertain which doses reach therapeutic PLs; and to find out which PLs are linked to better treatment outcomes and whether they involve worse tolerability, safety, or compliance.

## 2. Materials and Methods

An observational, prospective, one-year follow-up study was conducted on people over 18 years old with schizophrenia (ICD 10, F-20) who were seriously ill (defined as scoring ≥ 5 on the Clinical Global Impression-severity (CGI-S) scale), receiving treatment with stabilized doses of PP3M or ARIM for at least one year (in “steady-state” PLs) before study enrollment (N = 68). The decision to prescribe to patients ARIM or PP3M, or standard or high doses, had been made by the clinicians who were treating them.

Participants were included in two groups: “standard-dose” (licensed) and “high-dose” (above licensed: PP3M > 525 mg/3 months or ARIM > 400 mg/month). PLs were divided into therapeutic reference ranges (TRRs), or “standard range” (PP3M: 20–60 ng/mL; ARIM: 150–250 ng/mL) and “high range” (above TRRs). Of the 68 subjects, 22 were on PP3M standard doses, 21 on PP3M high doses, 12 on ARIM standard doses, and 13 on ARIM high doses. Patients were selected from each group randomly (stratified randomization).

The subjects’ gender, age, length of illness, previous AP treatments, weight, smoking habit, and concomitant medications were recorded. Patients treated with high doses were aiming to achieve clinical stabilization due to a lack of effectiveness after months of treatment with lower doses. PLs and the relationship between doses and PLs were determined. Treatment compliance and its relationship with dose and PLs were evaluated. To assess effectiveness, the CGI-S scale was administered (by the clinicians who treated the participants during the study, not by the study team members), and hospital admissions were recorded, comparing both with the previous year; the relationship of treatment outcomes with AP doses and plasma levels was also studied. The different doses and PLs, and changes in hospitalizations, CGI-S scores, and laboratory alterations, were compared to determine if a relationship exists between them. To assess tolerability and safety, reported adverse effects (AEs), and also weight, blood count, biochemistry, and prolactin levels, were collected. The relationship between both doses and PLs was also determined.

Blood extractions were performed after fasting at the lowest PLs, immediately before the administration of the next dose of either PP3M or ARIM. In all cases, the site of LAI administration was gluteal. Quantification of 9-hydroxyrisperidone and aripiprazole in serum was performed using liquid chromatography/mass tandem. To measure prolactin, participants had a 30 min rest before blood extraction. A specialized laboratory conducted the measurement of drug levels to ensure that levels were accurate.

A descriptive analysis of the sociodemographic and clinical variables of the sample was performed. For the analysis between treatment groups, χ2 was used for categorical variables and Student’s *t*-test for quantitative paired data. Blood level data were eligible for parametrical testing given that they followed a normal “gaussian” distribution. The confidence interval was established at 95%. For data processing, the “R Development Core Team” program (v 3.4.1) MASS Package (v 7.3-45) was used. In addition, considering the design as a cohort study (comparing the frequency of occurrence of the event “high plasma levels” between two groups, standard dose and high dose, with the latter considered the exposure group), the relative risk was calculated to see how many more times the event “high plasma levels” tended to occur in the high-dose group compared to the group not exposed (standard dose).

## 3. Results

The sample was made up mostly of middle-aged (48.1 [18.3]-year old) men (76.5%). All participants studied identified as male/male or female/female, with a 15.7 (7.9)-year length of illness. All of them had received prior antipsychotic treatment: 38 patients had been treated with oral antipsychotics (9 clozapine (578.6 [110.4] mg), 6 risperidone (7.9 [1.3] mg), 5 olanzapine (26.1 [7.2] mg), 7 aripiprazole (32.9 [9.4] mg), 5 paliperidone (12.3 [2.2] mg), and 6 with others); 30 patients had been treated with LAI antipsychotics (6 LAI risperidone (56.2 [6.3] mg/14 d), 11 LAI paliperidone (221.3 [30.1] mg/28 d, 9 LAI aripiprazole (706.9 [101.7] mg/28 d, and 4 with other LAI). There were no differences between the type of AP or dose related to these variables. No significant changes in weight or smoking habits during follow-up were found (Table 1).

**Figure 1:**
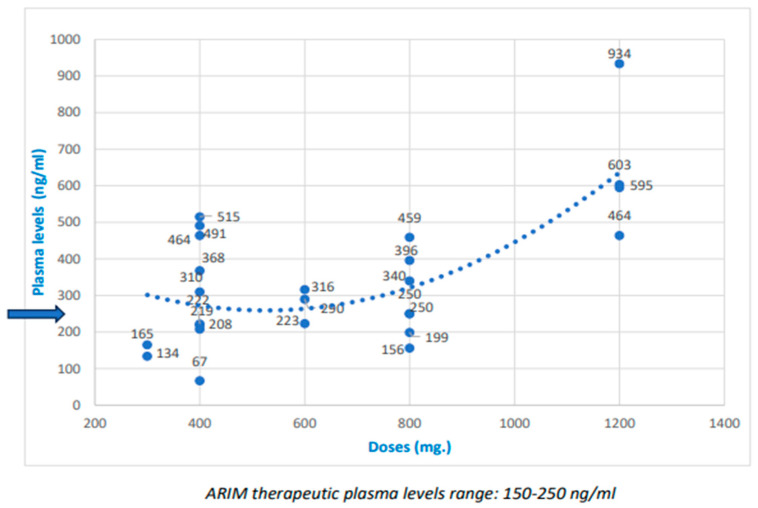
Doses and plasma levels of aripiprazole one-month (ARIM), and trend line (The blue arrow indicates the maximum therapeutic level).

**Figure 2:**
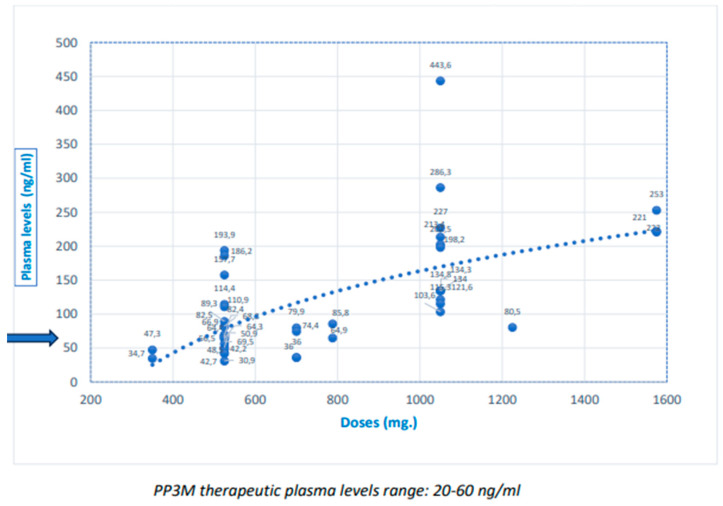
Doses and plasma levels of paliperidone tree-month (PP3M), and trend line (The blue arrow indicates the maximum therapeutic level).

The dose/PL ratio is shown in Table 2, with the dispersion of the dose values and PLs, and trend lines, in Figure 1 and Figure 2. Although some relationships do exist between ARIM and PP3M doses and plasma levels, they are not clearly linear. Figure 1 and Figure 2 show the trend line to judge this relationship. Indeed, the dose/PL correlation was not linear, especially with ARIM. In more than half of the subjects with standard doses, PLs above the TRR were reached (Table 3), mainly with PP3M. Some participants with high doses did not achieve high PLs, mainly with ARIM; in a few cases, standard doses did not achieve TRRs. The relative risk (RR) for “high PLs” in the “high dose” group compared to the “standard dose” group was approximately double (relative risk (RR) for high doses/high levels = 1.8; RR PP3M = 2.2; ARIM RR = 2.3).

All participants remained in the study regardless of the AP, the dose, or the PL. The severity of illness decreased after 12 months of follow-up, somewhat more frequently in subjects with greater severity, but they were mainly those receiving high doses. Hospitalizations decreased almost exclusively in those subjects with high plasma levels (Table 4). No significant differences (no relationships) were found between the concrete different doses and PLs, changes in hospitalizations, CGI-S scores, and laboratory alterations/side effects, so no PL was identified at which an improvement in these measures is seen (or no further improvement is seen).

More than 25% of people studied reported adverse effects, although none were serious or led to a change in AP. Side effects were not generally more frequent in the high-dose or in the high-PL groups (only for parkinsonism in patients on PP3M). The anticholinergic effects were related to concomitant medication use (*p* < 0.01) (Table 1). Alterations in laboratory tests (>20% over normal range) were found in almost two-thirds of participants, although without severity or a need to change treatment. The most common were elevations in lipids (found in 2/3 of participants) and prolactin (always in subjects with PP3M, and related to high levels; *p* < 0.05). Blood count, glucose, and hepatic function elevations were found in almost 20% of people (Table 1). These were not twice the upper limit of normal and they were not related to the kind of AP or to the dose (standard or high), although a few of them were related to high PLs. Both the percentage of patients with adverse effects and that of patients with laboratory test alterations decreased during follow-up.

Concomitant medications (psychotropic drugs) were prescribed in more than 50% of participants, with benzodiazepines, antidepressants, and other oral APs (mainly used as hypnotics) being the most common. The use of these medications decreased during the follow-up and was not linked to the AP used, doses, PLs, or outcomes (Table 1). Antiparkinsonian medications were used much more with PP3M, although without regard to dose or PLs.

No differences were found in these outcomes concerning gender, age, weight, or tobacco consumption (no significant relationships were encountered).

## 4. Discussion

### 4.1. Therapeutic Monitoring of SGA LAIs

SGA LAIs are a group of medications widely used in patients with schizophrenia and in a considerable number of complex cases at high doses [16,45,46,47,48,49,50,55]. For these reasons, awareness of the PLs achieved with the different doses prescribed and their relationship with the treatment outcomes of this profile of patients [16,46,47,48,49,52,53] is essential for optimizing their use [1,7,9,27,33,59], especially taking into account the lack of data [32,33,34,35,36,37,38,39].

Therapeutic drug monitoring has been proposed to estimate both response and toxicity in a given patient, but its usefulness has been called into question since studies with APs have yielded contradictory results [33,37,38,39,40,41], given the interindividual variability in pharmacokinetics [37,40,41,42]. In studies with seriously ill people, a curvilinear relationship was found between olanzapine doses and PLs, but not with risperidone [37,40,55,56]. It has also been observed that PLs vary due to factors such as sex, age, weight, dose, or smoking habit, but without explaining the differences in PLs between patients [41,60]. A greater correlation has been found between receptor occupancy and PLs than with the dose [36,37,40]. What seems clear is that PLs provide better therapeutic guidance than doses [36,37,38,39,40,41,42].

#### Doses and Plasma Levels of Aripiprazole and Paliperidone LAI

A retrospective evaluation of SGA-LAI levels found a surprisingly high incidence of PLs below the TRR in more than half of the subjects, despite maintaining the recommended dose, which implies that they may be underdosed in routine clinical practice [40]. Nevertheless, TRRs have been defined for oral preparations, and it is possible that for LAIs they may be different [32,33,34,35,36,37]. A meta-analysis of 20 APs exploring whether higher doses were appropriate for some people showed that doses higher than the licensed may, on average, not provide greater efficacy; for some APs, however, high doses could be tried because their dose–response curves did not stabilize [37,38,39]. In contrast, for APs with clearly increasing dose–response curves, high doses might be more effective [32,37,40,52,61]. Dose–response relationships in specific populations, such as treatment-resistant patients, are likely to differ [37,40].

A study with ARIM [62] found that the effective dose was 463 mg/4 weeks. Regarding paliperidone one-month, the effective dose was 120–150 mg/4 weeks [56,57,58]. In randomized studies evaluating pharmacokinetic profiles, higher peak PLs were found after dosing in the deltoid than in the gluteal [33,63]. A debate is open on whether LAI SGAs injected in the deltoid versus the gluteal muscle are therapeutically equivalent [64]. In our study, all participants were injected in the gluteal muscle, so this is not a possible cause of the different plasma levels reached.

Few studies have shown a correlation between dose and PLs with ARIM [41,62]. Our research did not find a clear linear correlation, and seems to indicate more of a correlation with PP3M than ARIM (Figure 1 and Figure 2). Moreover, the relative risk of achieving high PLs in the high-dose group was only about twice that of the standard-dose group.

The evidence of a relationship between PLs and clinical effectiveness and side effects is scarce [52,55,65], albeit under discussion [38,52]. Two studies found a relationship between increased ARIM PLs and antipsychotic response [41,51,66], as we found not only with ARIM but also with PP3M, while another reported better cognitive performance in subjects with high aripiprazole PLs [63]. On the other hand, high PLs are considered unlikely to increase adverse effects [41,48,51,52]

Regarding factors influencing aripiprazole levels, studies investigating age, gender, weight, and PLs did not show a clear correlation [41,60], and we did not find any relationship.

Regarding the influence of co-medications, studies have shown an increase after treatment with paroxetine, a decrease with carbamazepine and valproate, and no influence of escitalopram, haloperidol, or clozapine. They have also shown that simultaneous treatment with CYP3A4 inducers and CYP2D6 inhibitors did change PLs [40,41,51]. We found no influence of concomitant treatment with antidepressants and oral APs on PLs.

At any rate, the findings of this research question the linear kinetics of ARIM and PP3M in this profile of people. Indeed, in a notable percentage of cases, standard doses reached PLs higher than the TRRs, so most participants studied had PLs above the TRR, which has to be highlighted. It can thus be assumed that these people, all of whom were seriously ill, required PLs above the TRRs for clinical stabilization. It must be remembered that to support the rationale behind high-dose therapy, an argument is that an insufficient amount of antipsychotic might not have a sufficient effect due to individual pharmacokinetic differences [45,46,47,48,49].

### 4.2. Plasma Levels and Response (Treatment Compliance, Effectiveness, Tolerability, and Toxicity)

In our study, clinical severity, measured with the CGI-S, decreased after one year of follow-up. This decrease was more frequent in those participants with greater severity, but who were also those most frequently achieving high PLs. Moreover, the decrease in hospitalizations was almost exclusively in those patients with PLs above the TRR. Summing up, the effectiveness of high PLs was greater than with standard levels, without affecting therapeutic compliance, and the possible need for high PLs to achieve clinical improvement in many people with severe schizophrenia has to be pointed out.

Lastly, it is noteworthy that tolerability was good and, although more than 25% of subjects reported some AEs, none were serious or involved a need for a treatment change. Moreover, the tolerability and safety of high doses and PLs are similar to those of standard doses and levels (adverse effects were only higher in the group with high PLs of PP3M for Parkinsonism). Excessive changes in laboratory tests (considered to be >20% over the upper limit of normality, a very strict criterion) were found in almost three-quarters of participants, although without severity (none twice the upper limit of normal) or the need to change treatment. The elevation of lipids (cholesterol or triglycerides) was found mainly in patients with PP3M, regardless of the dose or PLs. Prolactin elevation was always linked to PP3M, and related to high PLs. This finding is not striking, given the well-known relationship between paliperidone and prolactin elevation (and even its decrease with aripiprazole) [51,52,67,68]. Concomitant medications have no influence on tolerability or safety.

These results raise the need to confirm the PLs achieved in clinical routine with the different doses prescribed, especially in people with clinical severity, poor adherence, and multiple relapses and hospitalizations. Furthermore, it should also be considered whether a good number of these patients require PLs above TRRs (possibly suitable only for less-severe populations or those without previous AP treatment failures), especially given that the tolerability of high PLs seems to be similar to the tolerability of standard (TRR) plasma levels.

Although the likelihood and intensity of most Aps’ adverse effects increase with dose, others are not dose-related [41,44,47,60,65]. While some studies have found more extrapyramidal side effects and elevated prolactin with high-dose SGA treatment than with standard doses, others have even reported less parkinsonism and fewer dropouts due to AEs [46,47,48,49,50]. Regarding aripiprazole, in one study subjects with higher PLs scored lower on an akathisia scale [65]. The available data do not provide conclusive evidence in this regard, and although several studies point to a good tolerability and safety of high-dose therapy [45,46,52,57], others did not show a clear association between PLs and AEs [50,51,52,67], a finding in line with our study.

In our study, there were no significant differences in safety or tolerability between standard and high PLs of both antipsychotics, which is important in order to test increases above standard doses to achieve effective levels and thus better clinical results without the risk of serious adverse effects [46,51,67,69] or reduced safety [70,71]. Moreover, the research shows that concomitant medications did not increase AEs.

We state that high plasma levels are linked to better treatment outcomes and without worse tolerability or safety. The most common alterations, as expected, were elevations in lipids and prolactin (this was significantly related to high PP3M PLs), but without clinical relevance.

### 4.3. Limitations and Strengths

Some limitations of this research need to be pointed out. This was an open-label study under pragmatic conditions. The study’s participants were already being treated with APs, so treatment was not assigned randomly but based on clinical criteria. This design has the risk that the two study groups (subjects with standard doses and subjects with high doses) are not homogeneous in terms of sociodemographic and clinical profiles. An attempt was made to minimize this potential bias by randomly selecting the participants from each cohort (stratified randomization).

In this study, we dichotomized our data into low and high doses/low and high PLs, instead of evaluating these as continuous variables, considering the methodological risks of this choice. We are aware of the fact that analyzing the data by running a linear regression/correlational analysis on them to determine if a relationship exists could be a valuable alternative. In any case, both analyses can help to propose a new TRR for ARIM and PP3M, based on identifying which PLs show improvements in hospitalization and CGI score, and also to determine at which PLs an increase in AEs is seen.

All the people in our study were rated as severely ill by the CGI-S, so the results may not be generalizable to populations that are not severely ill. Furthermore, the sample size was relatively small as it was conditioned by precisely this specific profile. Moreover, unmeasured factors related to the underlying clinical decision to treat a patient with ARIM or PP3M, or with standard or high doses, are a limitation of observational studies such as this.

We used the CGI-S to measure a clinically meaningful change in severity, which is a non-specific instrument and a potential limitation. No formal adverse-effect assessment scales were applied, with an ad hoc questionnaire designed by the study researchers for the evaluation of side effects used instead.

As for the strengths, our research provides a perspective of real-world results based on routine clinical practice, and it is the first study that measures the relationship of different doses with PLs of the most recently used LAI APs in people with severe schizophrenia. Moreover, it also assesses the treatment compliance, effectiveness, and tolerability of different doses and PLs. The number of PL quantifications of doses of these SGA LAIs above the licensed amount (half of the participants, 34) is also remarkable; to the best of our knowledge, there is no published study in this regard.

## 5. Conclusions

Given that AP treatment effectiveness is related, among other factors, to the achievement of optimal plasma levels, the search for the relationship between doses and PLs, and clinical response, is a step towards precision medicine in people with schizophrenia [72]. This is especially important for the most severe or “resistant” to treatment [73], who are frequently prescribed high doses to achieve clinical improvement, and also for the most recent LAI antipsychotics, which are increasingly used. The knowledge of these relationships can better guide practice and improve individualized treatment.

This study shows the PLs achieved with different doses and their relationship with the effectiveness and tolerability in patients with poor evolution. It was found that there was no clear linear relationship between doses of ARIM or PP3M and PLs; moreover, in many cases, standard doses, and not only high doses, achieved plasma levels above the TRR. In addition, improvements in treatment outcomes in people with severe schizophrenia were shown to be related to PLs above the established therapeutic ranges, without worse safety, tolerability, or compliance.

For this reason, one of the conclusions of the study is the proposal that the established TRRs for patients with clinical severity, multiple relapses, and previous antipsychotic treatment failures should be revised. These new TRRs could be related to those PLs that showed greater treatment effectiveness in our study. The data we have here could be used to help find what that revision should be. It is also necessary to remember that on the one hand, the TRRs were defined for oral preparations and may be different for LAIs, and on the other, the TRR values may be based more on North American than on European populations, as studied here.

Lastly, most of the study participants can be considered “treatment-resistant”, and were candidates for treatment with clozapine. Our findings make it possible to consider ARIM and PP3M, at PLs above the TRR, as a possible alternative for them, being safer, more tolerable, and more adherent [74].

Nevertheless, these preliminary findings have to be confirmed with studies with a larger number of subjects. It should also be determined whether they are related more to pharmacokinetic than to pharmacodynamic variables. If this is not the case, a subsequent genotyping study could be useful (e.g., with “drug response” research studying DRD3, CYP3D6, CYP219, and CYP1A2 genes) [75,76,77].

## Figures and Tables

**Table 1:** Sample clinical characteristics.

N: 68	Base/Previous Year	1-Year Follow-Up
CGI-S scale scores	5.4 (1.2)	4.7 (1.1) *
N# hospitalizations [X(SD)]	0.4 (0.2)	0.2 (0.1) **
Hospitalizations (% yes)	17.6	11.4 **
Weight kg. [X(SD)] ^$^	85.5 (21.3)	82.1 (18.6)
Tobacco use (% yes) ^$^	76.5	72.8
Concomitant medications (%). Any.	57.3	45.6 **
*Antidepressants*	34.2	29 *
*Mood stabilizers*	11.4	11.4
*Benzodiazepines*	38.2	34.2 *
*Other oral antipsychotics*	29	27.3
*Antiparkinsonians (Biperiden)*	38.2	35.3
*Other medications (with possible interactions)*	11.4	11.4
Laboratory test alterations (>20% normal range **ⱡ**) (%). Any.	68.4	57.3 **
*Blood count*	17.6	18.2
*Glucose*	18.2	19.1
*Lipids (cholesterol or triglycerides)*	68.4	60.8 *
*Hepatic function*	22.8	20.9
*Renal function*	2.2	2.2
*Cystatine C*	4.4	4.4
*Prolactin*	45.6 (57.3% HL)	38.2
Reported adverse effects (%). Any.	29	22.8 **
*Parkinsonism*	20.9 (91.2% PP3M)	19.1 *
*Sedation*	9.1	8.8
*Akathisia*	4.4	4.2
*Anticholinergic effects*	19.1	18.2
*Sexual disfunction*	18.2	17.6
*Other*	10.5	10.5
Blood pressure out of range (% yes)	9.5	9.1

X: mean; SD: standard deviation; HL: high levels; *: *p* < 0.05; **: *p* < 0.01. $: no differences between AP or dose; ⱡ: none twice the upper limit of normal.

**Table 2:** Doses and plasma levels of PP3M and ARIM.

N: 68	
**PP3M (N = 43)**	
Doses [X(SD)]	781.4 (114.2)
Plasma levels [X(SD)]	110.3 (32.1)
Plasma level/dose ratio	0.1
***PP3M standard doses (N = 22)***	
Doses [X(SD)]	511.5 (183.5)
Plasma levels [X(SD)]	41.6 (12.9)
Plasma level/dose ratio	0.1
***PP3M high doses (N = 21)***	
Doses [X(DE)]	2061.5 (412.1)
Plasma levels [X(SD)]	144 (22.6)
Plasma level/dose ratio	0.1
**ARIM (N = 25)**	
Doses [X(SD)]	656 (201.2)
Plasma levels [X(SD)]	345.5 (111.2)
Plasma level/dose ratio	0.5
***ARIM standard doses (N = 12)***	
Doses [X(SD)]	387.5 (87.8)
Plasma levels [X(SD)]	173.7 (44.5)
Plasma level/dose ratio	0.4
***ARIM high doses (N = 13)***	
Doses [X(SD)]	900 (189.6)
Plasma levels [X(SD)]	437.6 (91.8)
Plasma level/dose ratio	0.5

X: mean; SD: standard deviation; ARIM: aripiprazole one-month; PP3M: paliperidone three-month.

**Table 3:** ARIM and PP3M dose and plasma level relationships.

N = 68	Standard PLs (n = 20)	High PLs (n = 48)
Standard doses (n = 34) **^,$^	16	18
High doses (n = 34)	4	30
PP3M standard doses (n = 22) **	10	12
PP3M high doses (n = 21)	2	19
ARIM standard doses (n = 12) *	7	5
ARIM high doses (n = 13)	1	12

ARIM: aripiprazole one-month PP3M: paliperidone three-month PLs.: plasma levels *: *p* < 0.01, **: *p* < 0.001 No significant differences by gender, except ^$^: *p* < 0.01 for men.

**Table 4:** Doses, plasma levels, and response (effectiveness and toxicity). Number of subjects for which hospitalizations and CGI-S scores went down, and laboratory test alterations.

N = 68	↓ Hospitalizations (n = 13)	↓ CGI-S Scoring (n = 43)	Lab Alterations (n = 41)
Standard doses (n = 34)	5	18	21
High doses (n = 34)	8 *	25	20
Standard PLs (n = 20)	3	20	16
High PLs (n = 48)	10 **	23	25 *

*: *p* < 0.01; **: *p* < 0.001 ↓: diminution between baseline and 1-year follow-up; PLs.: plasma levels; Lab.: laboratory test. No significant differences by age or gender.

## Data Availability

Data are contained within the article.

## References

[1] Shad M.U. (2023). Seventy Years of Antipsychotic Development: A Critical Review. Biomedicines.

[2] Correll C.U., Rubio J.M., Kane J.M. (2018). What Is the Risk-Benefit Ratio of Long-Term Antipsychotic Treatment in People with Schizophrenia?. World Psychiatry.

[3] Ceraso A., Lin J.J., Schneider-Thoma J., Siafis S., Tardy M., Komossa K., Heres S., Kissling W., Davis J.M., Leucht S. (2020). Maintenance Treatment with Antipsychotic Drugs for Schizophrenia. Cochrane Database Syst. Rev..

[4] Meyer R., Skov K., Dhillon I.K., Olsson E., Graudal N.A., Baandrup L., Jürgens G. (2022). Onset of Action of Selected Second-Generation Antipsychotics (Pines)—A Systematic Review and Meta-Analyses. Biomedicines.

[5] Tiihonen J., Mittendorfer-Rutz E., Majak M., Mehtälä J., Hoti F., Jedenius E., Enkusson D., Leval A., Sermon J., Tanskanen A. (2017). Real-World Effectiveness of Antipsychotic Treatments in a Nationwide Cohort of 29,823 Patients With Schizophrenia. JAMA Psychiatry.

[6] López-Muñoz F., Tracy D.K., Povedano-Montero F.J., Breedvelt J., García-Pacios J., Fernández-Martín M.P., Rubio G., Álamo C. (2019). Trends in the Scientific Literature on Atypical Antipsychotic Drugs in the United Kingdom: A Bibliometric Study. Ther. Adv. Psychopharacol..

[7] Fernández-Miranda J.J., Díaz-Fernández S., López-Muñoz F. (2022). Effectiveness of More Personalized, Case-Managed, and Multicomponent Treatment for Patients with Severe Schizophrenia Compared to the Standard Treatment: A Ten-Year Follow-Up. J. Pers. Med..

[8] Kane J.M., Kishimoto T., Correll C.U. (2013). Non-Adherence to Medication in Patients with Psychotic Disorders: Epidemiology, Contributing Factors and Management Strategies. World Psychiatry.

[9] Díaz-Fernández S., López-Muñoz F., Fernández-Miranda J.J. (2021). Psychosocial and Pharmacological Approaches for Improving Treatment Adherence and Outcomes in People With Severe Schizophrenia: A 10-Year Follow-Up. J. Psychiatr. Pract..

[10] Bogers J.P.A.M., Hambarian G., Walburgh Schmidt N., Vermeulen J.M., de Haan L. (2022). Risk Factors for Psychotic Relapse after Dose Reduction or Discontinuation of Antipsychotics in Patients with Chronic Schizophrenia. A Meta-Analysis of Randomized Controlled Trials. Schizophr. Bull..

[11] Auxilia A.M., Buoli M., Caldiroli A., Carnevali G.S., Tringali A., Nava R., Clerici M., Capuzzi E. (2023). High Rate of Discontinuation during Long-Acting Injectable Antipsychotic Treatment in Patients with Psychotic Disorders. Biomedicines.

[12] Huang C.-Y., Fang S.-C., Shao Y.-H.J. (2021). Comparison of Long-Acting Injectable Antipsychotics with Oral Antipsychotics and Suicide and All-Cause Mortality in Patients with Newly Diagnosed Schizophrenia. JAMA Netw. Open.

[13] Pompili M., Orsolini L., Lamis D.A., Goldsmith D.R., Nardella A., Falcone G., Corigliano V., Luciano M., Fiorillo A. (2017). Suicide Prevention in Schizophrenia: Do Long-Acting Injectable Antipsychotics (LAIs) Have a Role?. CNS Neurol. Disord. Drug Targets.

[14] Díaz-Fernández S., Frías-Ortiz D.F., Fernández-Miranda J.J. (2020). Suicide Attempts in People with Schizophrenia before and after Participating in an Intensive Case Managed Community Program: A 20-Year Follow-Up. Psychiatry Res..

[15] Kishimoto T., Hagi K., Nitta M., Kane J.M., Correll C.U. (2019). Long-Term Effectiveness of Oral Second-Generation Antipsychotics in Patients with Schizophrenia and Related Disorders: A Systematic Review and Meta-Analysis of Direct Head-to-Head Comparisons. World Psychiatry.

[16] Fernández-Miranda J.J., Díaz-Fernández S., López-Muñoz F. (2022). The Use of Second-Generation Antipsychotics in Patients with Severe Schizophrenia in the Real World: The Role of the Route of Administration and Dosage-A 5-Year Follow-Up. Biomedicines.

[17] Correll C.U., Citrome L., Haddad P.M., Lauriello J., Olfson M., Calloway S.M., Kane J.M. (2016). The Use of Long-Acting Injectable Antipsychotics in Schizophrenia: Evaluating the Evidence. J. Clin. Psychiatry.

[18] Kishimoto T., Hagi K., Kurokawa S., Kane J.M., Correll C.U. (2021). Long-Acting Injectable versus Oral Antipsychotics for the Maintenance Treatment of Schizophrenia: A Systematic Review and Comparative Meta-Analysis of Randomised, Cohort, and Pre-Post Studies. Lancet Psychiatry.

[19] Lin D., Thompson-Leduc P., Ghelerter I., Nguyen H., Lafeuille M.-H., Benson C., Mavros P., Lefebvre P. (2021). Real-World Evidence of the Clinical and Economic Impact of Long-Acting Injectable Versus Oral Antipsychotics Among Patients with Schizophrenia in the United States: A Systematic Review and Meta-Analysis. CNS Drugs.

[20] Fernández-Miranda J.J., Díaz-Fernández S., López-Muñoz F. (2021). Oral versus Long-Acting Injectable Antipsychotic Treatment for People with Severe Schizophrenia: A 5-Year Follow-up of Effectiveness. J. Nerv. Ment. Dis..

[21] García-Carmona J.A., Simal-Aguado J., Campos-Navarro M.P., Valdivia-Muñoz F., Galindo-Tovar A. (2021). Evaluation of Long-Acting Injectable Antipsychotics with the Corresponding Oral Formulation in a Cohort of Patients with Schizophrenia: A Real-World Study in Spain. Int. Clin. Psychopharmacol..

[22] Ostuzzi G., Bertolini F., Tedeschi F., Vita G., Brambilla P., Del Fabro L., Gastaldon C., Papola D., Purgato M., Nosari G. (2022). Oral and Long-Acting Antipsychotics for Relapse Prevention in Schizophrenia-Spectrum Disorders: A Network Meta-Analysis of 92 Randomized Trials Including 22,645 Participants. World Psychiatry.

[23] Taipale H., Mehtälä J., Tanskanen A., Tiihonen J. (2018). Comparative Effectiveness of Antipsychotic Drugs for Rehospitalization in Schizophrenia-A Nationwide Study with 20-Year Follow-Up. Schizophr. Bull..

[24] Rubio J.M., Schoretsanitis G., John M., Tiihonen J., Taipale H., Guinart D., Malhotra A.K., Correll C.U., Kane J.M. (2020). Psychosis Relapse during Treatment with Long-Acting Injectable Antipsychotics in Individuals with Schizophrenia-Spectrum Disorders: An Individual Participant Data Meta-Analysis. Lancet Psychiatry.

[25] Kane J.M., Schooler N.R., Marcy P., Correll C.U., Achtyes E.D., Gibbons R.D., Robinson D.G. (2020). Effect of Long-Acting Injectable Antipsychotics vs Usual Care on Time to First Hospitalization in Early-Phase Schizophrenia: A Randomized Clinical Trial. JAMA Psychiatry.

[26] Díaz-Fernández S., Frías-Ortiz D.F., Fernández-Miranda J.J. (2022). Mirror Image Study (10 Years of Follow-up and 10 of Standard Pre-Treatment) of Psychiatric Hospitalizations of Patients with Severe Schizophrenia Treated in a Community-Based, Case-Managed Programme. Rev. Psiquiatr. Salud Ment. (Engl. Ed.).

[27] Nasrallah H.A. (2018). Triple Advantages of Injectable Long Acting Second Generation Antipsychotics: Relapse Prevention, Neuroprotection, and Lower Mortality. Schizophr. Res..

[28] Fu A.Z., Pesa J.A., Lakey S., Benson C. (2022). Healthcare Resource Utilization and Costs before and after Long-Acting Injectable Antipsychotic Initiation in Commercially Insured Young Adults with Schizophrenia. BMC Psychiatry.

[29] Paton C., Okocha C.I., Patel M.X. (2022). Can the Use of Long-Acting Injectable Antipsychotic Preparations Be Increased in Routine Clinical Practice and the Benefits Realised?. Ther. Adv. Psychopharmacol..

[30] Bartoli F., Cavaleri D., Callovini T., Riboldi I., Crocamo C., D’Agostino A., Martinotti G., Bertolini F., Ostuzzi G., Barbui C. (2022). Comparing 1-Year Effectiveness and Acceptability of Once-Monthly Paliperidone Palmitate and Aripiprazole Monohydrate for Schizophrenia Spectrum Disorders: Findings from the STAR Network Depot Study. Psychiatry Res..

[31] Mason K., Barnett J., Pappa S. (2021). Effectiveness of 2-Year Treatment with Aripiprazole Long-Acting Injectable and Comparison with Paliperidone Palmitate. Ther. Adv. Psychopharmacol..

[32] McCutcheon R., Beck K., D’Ambrosio E., Donocik J., Gobjila C., Jauhar S., Kaar S., Pillinger T., Reis Marques T., Rogdaki M. (2018). Antipsychotic Plasma Levels in the Assessment of Poor Treatment Response in Schizophrenia. Acta Psychiatr. Scand..

[33] Schoretsanitis G., Kane J.M., Correll C.U., Marder S.R., Citrome L., Newcomer J.W., Robinson D.G., Goff D.C., Kelly D.L., Freudenreich O. (2020). Blood Levels to Optimize Antipsychotic Treatment in Clinical Practice. J. Clin. Psychiatry.

[34] Meyer J.N., Stahl S.M. (2021). The Clinical Use of Antipsychotic Plasma Levels. The Clinical Use of Antipsychotic Plasma Levels.

[35] Sheehan J.J., Reilly K.R., Fu D.-J., Alphs L. (2012). Comparison of the Peak-to-Trough Fluctuation in Plasma Concentration of Long-Acting Injectable Antipsychotics and Their Oral Equivalents. Innov. Clin. Neurosci..

[36] Hiemke C., Bergemann N., Clement H., Conca A., Deckert J., Domschke K., Eckermann G., Egberts K., Gerlach M., Greiner C. (2018). Consensus Guidelines for Therapeutic Drug Monitoring in Neuropsychopharmacology: Update 2017. Pharmacopsychiatry.

[37] Leucht S., Crippa A., Siafis S., Patel M.X., Orsini N., Davis J.M. (2020). Dose-Response Meta-Analysis of Antipsychotic Drugs for Acute Schizophrenia. Am. J. Psychiatry.

[38] Correll C.U., Kim E., Sliwa J.K., Hamm W., Gopal S., Mathews M., Venkatasubramanian R., Saklad S.R. (2021). Pharmacokinetic Characteristics of Long-Acting Injectable Antipsychotics for Schizophrenia: An Overview. CNS Drugs.

[39] Schoretsanitis G., Baumann P., Conca A., Dietmaier O., Giupponi G., Gründer G., Hahn M., Hart X., Havemann-Reinecke U., Hefner G. (2021). Therapeutic Drug Monitoring of Long-Acting Injectable Antipsychotic Drugs. Ther. Drug Monit..

[40] Hýža M., Šilhán P., Češková E., Skřont T., Kacířová I., Uřinovská R., Grundmann M. (2021). Plasma Levels of Long-Acting Injectable Antipsychotics in Outpatient Care: A Retrospective Analysis. Neuropsychiatr. Dis. Treat..

[41] Hart X.M., Eichentopf L., Lense X., Riemer T., Wesner K., Hiemke C., Gründer G. (2021). Therapeutic Reference Ranges for Psychotropic Drugs: A Protocol for Systematic Reviews. Front. Psychiatry.

[42] Gardner D.M., Murphy A.L., O’Donnell H., Centorrino F., Baldessarini R.J. (2010). International Consensus Study of Antipsychotic Dosing. Am. J. Psychiatry.

[43] Canada’s Drug and Health Technology Agency (CADTH) (2011). Systematic Review of Combination and High Dose AAPs for Schizophrenia.

[44] (2014). Royal College of Psychiatrists Consensus Statement on High-Dose Antipsychotic Medication.

[45] Sommer I.E., Begemann M.J.H., Temmerman A., Leucht S. (2012). Pharmacological Augmentation Strategies for Schizophrenia Patients with Insufficient Response to Clozapine: A Quantitative Literature Review. Schizophr. Bull..

[46] Fernández-Miranda J.J., Díaz-Fernández S., López-Muñoz F. (2020). High Doses of Second-Generation Long-Acting Antipsychotics in the Treatment of Patients with Severe Resistant Schizophrenia. A Six-Year Mirror-Image Study. Psychiatry Clin. Psychopharmacol..

[47] Meltzer H.Y., Bobo W.V., Roy A., Jayathilake K., Aygun C., Elif E.A., Yagcioglu A., Small J.G. (2008). A Randomized, Double-Blind Comparison of Clozapine and High-Dose Olanzapine in Treatment-Resistant Patients With Schizophrenia. J. Clin. Psychiatry.

[48] Fernández-Miranda J.J., Díaz-Fernández S. (2017). Tolerability of Effective High Doses of Paliperidone Palmitate in Patients with Severe Resistant Schizophrenia. Int. Clin. Psychopharmacol..

[49] Meltzer H.Y.Y., Lindenmayer J.-P.P., Kwentus J., Share D.B.B., Johnson R., Jayathilake K. (2014). A Six Month Randomized Controlled Trial of Long Acting Injectable Risperidone 50 and 100mg in Treatment Resistant Schizophrenia. Schizophr. Res..

[50] Fernández-Miranda J.J., Caramés-García V., Sánchez-García A. (2015). Effectiveness, Good Tolerability, and High Compliance of Doses of Risperidone Long-Acting Injectable Higher Than 75 Mg in People with Severe Schizophrenia: A 3-Year Follow-Up. J. Clin. Psychopharmacol..

[51] Correll C.U., Howes O.D. (2021). Treatment-Resistant Schizophrenia: Definition, Predictors, and Therapy Options. J. Clin. Psychiatry.

[52] Lin S.-K., Chen C.-K., Liu Y.-L. (2011). Aripiprazole and Dehydroaripiprazole Plasma Concentrations and Clinical Responses in Patients with Schizophrenia. J. Clin. Psychopharmacol..

[53] Fernández-Miranda J.J., Díaz-Fernández S., López-Muñoz F. (2021). Adherence, Tolerability and Effective Doses of Aripiprazole Once-Monthly in the Long-Term Treatment of Patients with Severe Schizophrenia. Curr. Pharm. Des..

[54] Salzman P.M., Raoufinia A., Legacy S., Such P., Eramo A. (2017). Plasma Concentrations and Dosing of 2 Long-Acting Injectable Formulations of Aripiprazole. Neuropsychiatr. Dis. Treat..

[55] Schoretsanitis G., Spina E., Hiemke C., de Leon J. (2018). A Systematic Review and Combined Analysis of Therapeutic Drug Monitoring Studies for Oral Paliperidone. Expert Rev. Clin. Pharmacol..

[56] Pandina G.J., Lindenmayer J.-P., Lull J., Lim P., Gopal S., Herben V., Kusumakar V., Yuen E., Palumbo J. (2010). A Randomized, Placebo-Controlled Study to Assess the Efficacy and Safety of 3 Doses of Paliperidone Palmitate in Adults with Acutely Exacerbated Schizophrenia. J. Clin. Psychopharmacol..

[57] Fernández-Miranda J.J., Díaz-Fernández S., De Berardis D., López-Muñoz F. (2021). Paliperidone Palmitate Every Three Months (PP3M) 2-Year Treatment Compliance, Effectiveness and Satisfaction Compared with Paliperidone Palmitate-Monthly (PP1M) in People with Severe Schizophrenia. J. Clin. Med..

[58] Mathews M., Gopal S., Singh A., Nuamah I., Pungor K., Tan W., Soares B., Kim E., Savitz A.J. (2020). Comparison of Relapse Prevention with 3 Different Paliperidone Formulations in Patients with Schizophrenia Continuing versus Discontinuing Active Antipsychotic Treatment: A Post-Hoc Analysis of 3 Similarly Designed Randomized Studies. Neuropsychiatr. Dis. Treat..

[59] Nakajima N., Mizoe N., Misawa F., Yamashita T., So R., Kitagawa K., Tanimoto K., Kishi Y., Fujii Y., Takeuchi H. (2021). Longitudinal Changes in Antipsychotic Dose in Patients Treated with Long-Acting Injectable Second-Generation Antipsychotics. Int. Clin. Psychopharmacol..

[60] Hoekstra S., Bartz-Johannessen C., Sinkeviciute I., Reitan S.K., Kroken R.A., Løberg E.-M., Larsen T.K., Rettenbacher M., Johnsen E., Sommer I.E. (2021). Sex Differences in Antipsychotic Efficacy and Side Effects in Schizophrenia Spectrum Disorder: Results from the BeSt InTro Study. NPJ Schizophr..

[61] Hart X.M., Hiemke C., Eichentopf L., Lense X.M., Clement H.W., Conca A., Faltraco F., Florio V., Grüner J., Havemann-Reinecke U. (2022). Therapeutic Reference Range for Aripiprazole in Schizophrenia Revised: A Systematic Review and Metaanalysis. Psychopharmacology.

[62] Meltzer H.Y., Risinger R., Nasrallah H.A., Du Y., Zummo J., Corey L., Bose A., Stankovic S., Silverman B.L., Ehrich E.W. (2015). A Randomized, Double-Blind, Placebo-Controlled Trial of Aripiprazole Lauroxil in Acute Exacerbation of Schizophrenia. J. Clin. Psychiatry.

[63] Hard M.L., Wehr A., von Moltke L., Du Y., Farwick S., Walling D.P., Sonnenberg J. (2019). Pharmacokinetics and Safety of Deltoid or Gluteal Injection of Aripiprazole Lauroxil NanoCrystal^®^ Dispersion Used for Initiation of the Long-Acting Antipsychotic Aripiprazole Lauroxil. Ther. Adv. Psychopharmacol..

[64] Yin J., Collier A.C., Barr A.M., Honer W.G., Procyshyn R.M. (2015). Paliperidone Palmitate Long-Acting Injectable Given Intramuscularly in the Deltoid Versus the Gluteal Muscle. J. Clin. Psychopharmacol..

[65] Hwang T.-J., Lo W.-M., Chan H.-Y., Lin C.-F., Hsieh M.H., Liu C.-C., Liu C.-M., Hwu H.-G., Kuo C.-H., Chen W.J. (2015). Fast Versus Slow Strategy of Switching Patients with Schizophrenia to Aripiprazole From Other Antipsychotics. J. Clin. Psychopharmacol..

[66] Weiden P.J., Du Y., von Moltke L., Wehr A., Hard M., Marandi M., Walling D.P. (2020). Pharmacokinetics, Safety, and Tolerability of a 2-Month Dose Interval Regimen of the Long-Acting Injectable Antipsychotic Aripiprazole Lauroxil: Results From a 44-Week Phase I Study. CNS Drugs.

[67] Misawa F., Kishimoto T., Hagi K., Kane J.M., Correll C.U. (2016). Safety and Tolerability of Long-Acting Injectable versus Oral Antipsychotics: A Meta-Analysis of Randomized Controlled Studies Comparing the Same Antipsychotics. Schizophr. Res..

[68] Osser D.N. (2017). Prolactin Monitoring in First-Episode Psychotic Patients. Schizophr. Res..

[69] Samara M.T., Dold M., Gianatsi M., Nikolakopoulou A., Helfer B., Salanti G., Leucht S. (2016). Efficacy, Acceptability, and Tolerability of Antipsychotics in Treatment-Resistant Schizophrenia. JAMA Psychiatry.

[70] Kishi T., Matsunaga S., Iwata N. (2016). Mortality Risk Associated with Long-Acting Injectable Antipsychotics: A Systematic Review and Meta-Analyses of Randomized Controlled Trials. Schizophr. Bull..

[71] Correll C.U., Solmi M., Croatto G., Schneider L.K., Rohani-Montez S.C., Fairley L., Smith N., Bitter I., Gorwood P., Taipale H. (2022). Mortality in People with Schizophrenia: A Systematic Review and Meta-Analysis of Relative Risk and Aggravating or Attenuating Factors. World Psychiatry.

[72] Wang X., Raoufinia A., Bihorel S., Passarell J., Mallikaarjun S., Phillips L. (2022). Population Pharmacokinetic Modeling and Exposure-Response Analysis for Aripiprazole Once Monthly in Subjects with Schizophrenia. Clin. Pharmacol. Drug Dev..

[73] Siskind D., Orr S., Sinha S., Yu O., Brijball B., Warren N., MacCabe J.H., Smart S.E., Kisely S. (2022). Rates of Treatment-Resistant Schizophrenia from First-Episode Cohorts: Systematic Review and Meta-Analysis. Br. J. Psychiatry.

[74] Martínez-Andrés J.A., García-Carmona J.A. (2020). Switching from Clozapine to Paliperidone Palmitate-3-Monthly Improved Obesity, Hyperglycemia and Dyslipidemia Lowering Antipsychotic Dose Equivalents in a Treatment-Resistant Schizophrenia Cohort. Int. Clin. Psychopharmacol..

[75] Kaneko H., Miura I., Kanno-Nozaki K., Horikoshi S., Hino M., Yabe H. (2018). COMT Val 108/158 Met Polymorphism and Treatment Response to Aripiprazole in Patients with Acute Schizophrenia. Neuropsychiatr. Dis. Treat..

[76] Zhang L., Brown S.J., Shan Y., Lee A.M., Allen J.D., Eum S., de Leon J., Bishop J.R. (2020). CYP2D6 Genetic Polymorphisms and Risperidone Pharmacokinetics: A Systematic Review and Meta-Analysis. Pharmacotherapy.

[77] Płaza O., Gałecki P., Orzechowska A., Gałecka M., Sobolewska-Nowak J., Szulc A. (2022). Pharmacogenetics and Schizophrenia—Can Genomics Improve the Treatment with Second-Generation Antipsychotics?. Biomedicines.

